# High-resolution analysis of germ cells from men with sex chromosomal aneuploidies reveals normal transcriptome but impaired imprinting

**DOI:** 10.1186/s13148-019-0720-3

**Published:** 2019-08-28

**Authors:** Sandra Laurentino, Laura Heckmann, Sara Di Persio, Xiaolin Li, Gerd Meyer zu Hörste, Joachim Wistuba, Jann-Frederik Cremers, Jörg Gromoll, Sabine Kliesch, Stefan Schlatt, Nina Neuhaus

**Affiliations:** 1 Centre of Reproductive Medicine and Andrology, University of Münster, Domagkstrasse 11, 48149 Münster, Germany; 2 Centre of Reproductive Medicine and Andrology, Department of Clinical and Surgical Andrology, University of Münster, Münster, Germany; 3 Department of Neurology, Institute of Translational Neurology, University Hospital of Münster, Münster, Germany

**Keywords:** Deep bisulfite sequencing, DNA methylation, Klinefelter syndrome, Male germline, Sex chromosome aneuploidy, Single-cell analysis, Sperm, Spermatogonia

## Abstract

**Background:**

The most common sex chromosomal aneuploidy in males is Klinefelter syndrome, which is characterized by at least one supernumerary X chromosome. While these men have long been considered infertile, focal spermatogenesis can be observed in some patients, and sperm can be surgically retrieved and used for artificial reproductive techniques. Although these gametes can be used for fertility treatments, little is known about the molecular biology of the germline in Klinefelter men. Specifically, it is unclear if germ cells in Klinefelter syndrome correctly establish the androgenetic DNA methylation profile and transcriptome. This is due to the low number of germ cells in the Klinefelter testes available for analysis.

**Results:**

Here, we overcame these difficulties and successfully investigated the epigenetic and transcriptional profiles of germ cells in Klinefelter patients employing deep bisulfite sequencing and single-cell RNA sequencing. On the transcriptional level, the germ cells from Klinefelter men clustered together with the differentiation stages of normal spermatogenesis. Klinefelter germ cells showed a normal DNA methylation profile of selected germ cell-specific markers compared with spermatogonia and sperm from men with normal spermatogenesis. However, germ cells from Klinefelter patients showed variations in the DNA methylation of imprinted regions.

**Conclusions:**

These data indicate that Klinefelter germ cells have a normal transcriptome but might present aberrant imprinting, showing impairment in germ cell development that goes beyond mere germ cell loss.

**Electronic supplementary material:**

The online version of this article (10.1186/s13148-019-0720-3) contains supplementary material, which is available to authorized users.

## Background

Klinefelter syndrome (KS) is the most common type of sex chromosomal aneuploidy in men, with a prevalence of 0.1–0.2% in the male population [1]. The majority of patients with KS present with a non-mosaic 47,XXY karyotype; however, 48,XXXY and 48,XXYY have also been described and are associated with a broad phenotypic spectrum [2]. The common phenotypic characteristic of individuals carrying extra X chromosomes is small testes accompanied in the majority of cases by the absence of sperm in the ejaculate (azoospermia) and endocrine changes (high gonadotropins, low to normal testosterone serum levels) [3]. Histological analyses revealed that the low numbers of sperm in the adult testis are the result of germ cell loss early during the development. In fact, numbers of spermatogonia are already greatly diminished in testicular tissues of prepubertal [4] as well as peri-pubertal boys [5]. Nevertheless, subpopulations of residual spermatogonia maintain the ability to differentiate into sperm, as demonstrated by studies evaluating testicular sperm extraction (TESE) in Klinefelter individuals of different ages. Success rates are highest in young men (15–19 years of age) with a 45% chance to isolate sperm [6]. Analyses of a total of 977 sperm from Klinefelter men (*n* = 10) by fluorescence in situ hybridization analysis demonstrated that 94.7% of cells were euploid compared to 99.4% in control samples [7]. Importantly, these aneuploidy rates in Klinefelter sperm were similar compared to those from patients with non-obstructive azoospermia [7]. Analysis of children born to men with non-mosaic KS after testicular sperm extraction and intracytoplasmic sperm injection revealed normal karyotypes in cohorts of 16 and 17 children, respectively [8, 9]. These euploid sperms likely originate from focal populations of euploid spermatogonia amidst XXY Sertoli cells [10]. Available data therefore suggests that the risk of transmission of chromosomal aneuploidy through the germline may be rather low.

However, different molecular mechanisms are being investigated to elucidate the specific biological consequences of the supernumerary sex chromosomes on phenotypes of Klinefelter patients, such as improper X chromosome inactivation and gene dosage effects [11]. X chromosome inactivation ensures compensation of X chromosomal gene dosage between the sexes and is controlled by the expression of the X-encoded long non-coding RNA XIST (X-inactive-specific transcript), which upon expression coats the X chromosome and thereby leads to silencing of the majority of genes [12, 13]. *XIST* expression appears to be regulated by methylation of a CpG island [14, 15]. Lymphocytes obtained from euploid 46,XY men show 100% methylation levels of selected CpG sites, whereas methylation levels in 46,XX females were at about 50% [16]. This is in line with the expected repression of *XIST* in 46,XY cells and the monoallelic expression in 46,XX cells, respectively. Consistent with this, XIST was found to be upregulated in the testis of Klinefelter patients compared to cellularity-matched controls [17]. Moreover, lymphocytes from Klinefelter patients showed *XIST* methylation levels comparable to females, which is in accordance with the presence of more than one X chromosome [16]. Apart from this, global changes in the methylation signature of leucocytes have been described in Klinefelter patients [18–20], suggesting that genome-wide changes in DNA methylation may also constitute a mechanism affecting Klinefelter phenotypes.

Whether the sperm of Klinefelter men is similarly affected by the changes in DNA methylation remains hitherto unknown as the number of sperm that can be obtained from these men is usually too low to perform such analyses. Importantly though, a number of studies have reported an association between male infertility and aberrant sperm DNA methylation for the genomic imprinted regions *H19*, *MEST*, and *SNRPN* [21]. These findings are of clinical relevance as the use of sperm with aberrant methylation profiles has been suggested to contribute to specific diseases in the offspring, which are more prevalent in children conceived through assisted reproductive technologies [22, 23].

In line with this, pleas for further research on the roles of ‘specific genes and of epigenetics’ were formulated in the framework of the International Workshop on the Klinefelter syndrome [24]. This need for analysis of Klinefelter germ cells has further grown as specialized centres routinely offer TESE with subsequent cryopreservation of sperm to pubertal and adult Klinefelter patients as means for fertility preservation. Studies of germ cells from Klinefelter men have been hampered so far by the scarcity of tissues and the extremely limited number of germ cells remaining in testes of KS men. Hence, we aimed at investigating the DNA methylation profiles using single-allele resolution analyses focusing on imprinted genes, germ cell marker genes, and *XIST* and the transcriptional profiles of germ cells in Klinefelter men employing single-cell analyses.

## Results

### Presence of germ cells in Klinefelter patient’s testes is detectable by morphology and immunochemical detection of VASA/DDX4

**Fig. 1:**
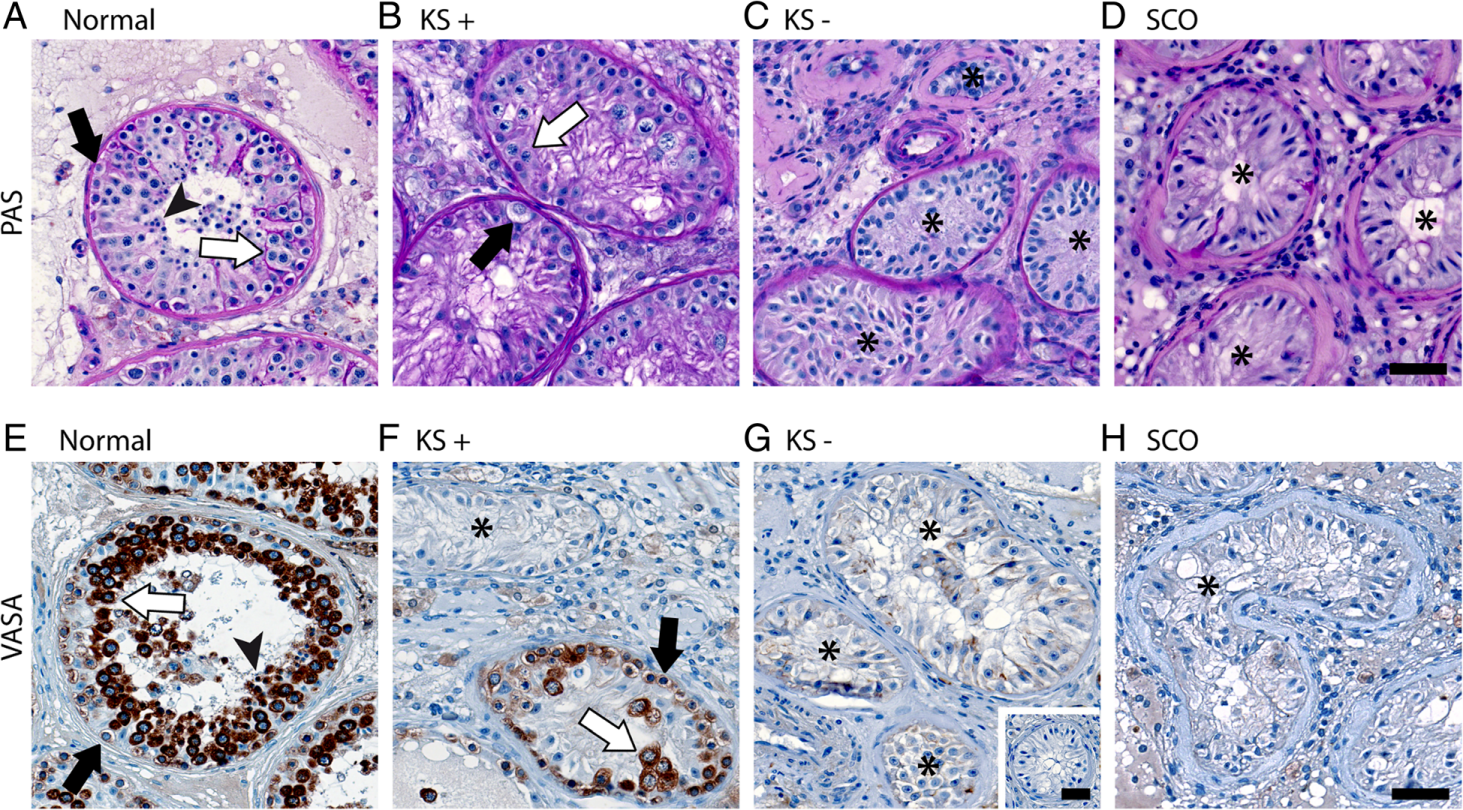
Micrographs demonstrating the presence of germ cells in testicular tissues of selected Klinefelter patients. **a**–**d** show PAS-stained sections and **e–h** depict sections following immunohistochemical stainings for VASA/DDX4. Germ cell types within the seminiferous tubules are indicated using black arrows for spermatogonia, white arrows for spermatocytes, and arrow heads for spermatids. Seminiferous tubules devoid of germ cells are marked by asterisks. Normal qualitatively normal spermatogenesis, KS Klinefelter syndrome, + with germ cells, − without germ cells, SCO Sertoli cell-only. Scale bars represent 50 μm

Klinefelter testicular tissue samples with and without germ cells were identified by histological analysis following PAS staining (Fig. 1a–d) and immunohistochemical detection of the germ cell marker VASA/DDX4 (Fig. 1e–h). Individual tubules showing focal spermatogenesis could be detected in selected Klinefelter tissues (Fig. 1b, f). This was in contrast to control samples with qualitatively normal spermatogenesis (Fig. 1a, e), which showed spermatogenesis in the majority of seminiferous tubules and samples with the complete absence of germ cells displaying a Sertoli cell-only phenotype (Fig. 1d, h).

### Germ cells at different stages of differentiation display normal transcriptome in Klinefelter testis

Using testicular tissues with qualitatively normal spermatogenesis, germ cells were enriched from testicular tissues using a differential plating approach. Transcriptional profiling was performed for selected marker genes using qPCR. Successful separation of germ cells from testicular somatic cells could be demonstrated by significantly higher expression of six germ cell marker genes (Additional file 1: Figure S1; *FGFR3*, *UTF1*, *RHOXF2/2B*, *MAGEA4*, *VASA/DDX4*, *RHOXF1*).

The same differential plating approach was applied to testicular samples from patients with KS. As the qPCR approach did not have sufficient resolution and sensitivity to enable the detection of germ cell-specific markers in such a small proportion of germ cells as present in Klinefelter tissues, subsequent transcriptional analysis was based on single-cell RNA-Seq of individual testicular cells obtained from one KS patient biopsy with germ cells.

**Fig. 2:**
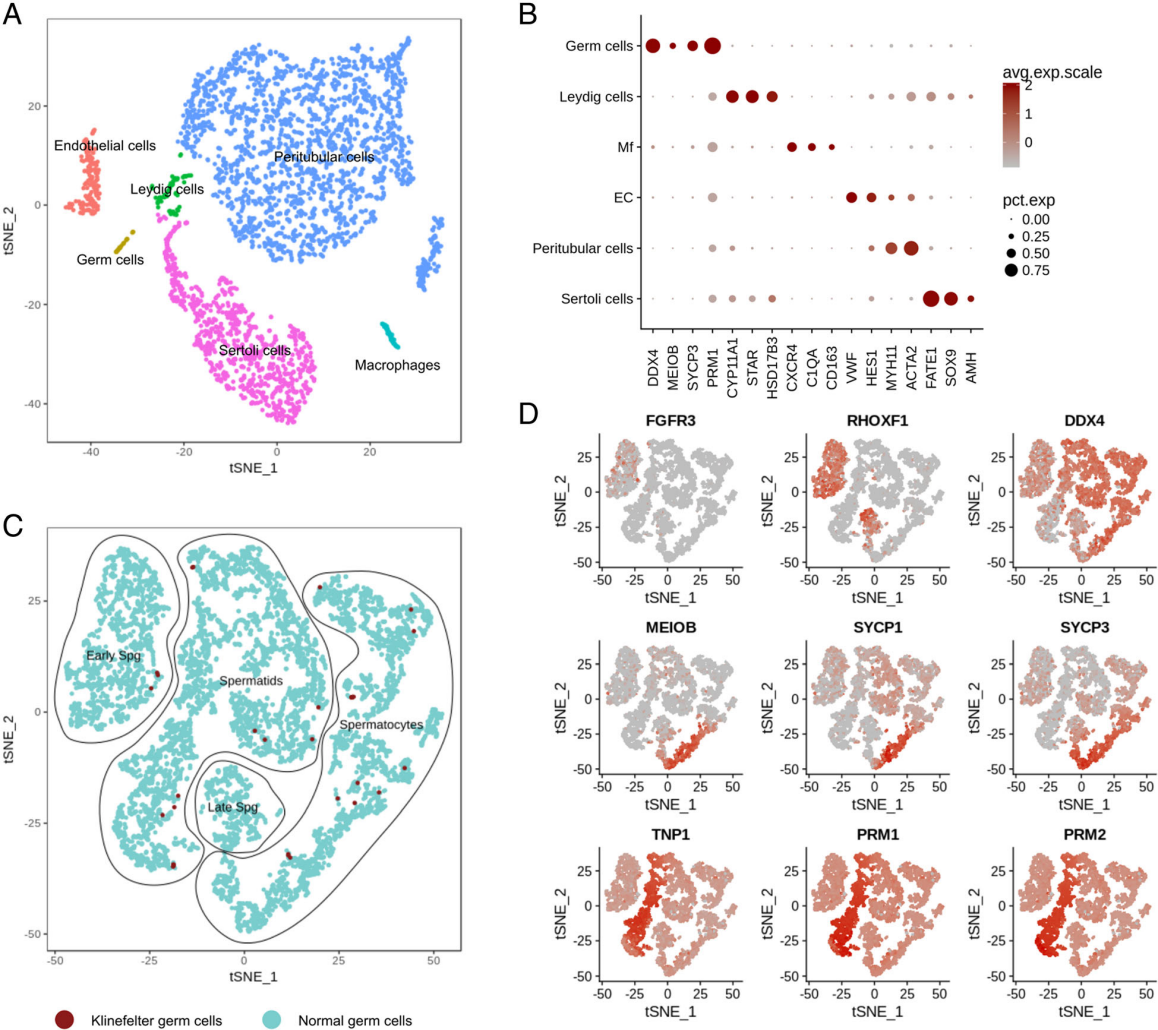
Single-cell analysis of Klinefelter testicular tissue and comparison with normal human testis transcriptome. **a** t-SNE plot showing the clustering of the different testicular cell types present in a Klinefelter sample. **b** Expression profiles of specific testicular cell marker genes in the different cell clusters identified in **a**. **c** t-SNE plot of germ cells from the Klinefelter patients and three men with full spermatogenesis (data obtained from [25]) clustered together. Relative expression of representative genes of germ cell differentiation stages are projected onto the t-SNE plot (**d**). Increased expression levels are depicted by increased red colour intensity. Upper panel represents spermatogonial (*FGFR3* and *RHOXF1*) and pan-germ cell marker *VASA/DDX4*, middle panel shows meiotic markers, and lower panel represents spermatid markers. Mf macrophages, EC endothelial cells, Spg spermatogonia, avg. exp. scale average expression scale, pct. exp percentage of cells expressing the marker

After filtering and quality control, 3289 cells were used for further analysis. Clustering was performed based on the use of published two to four specific cell markers (Fig. 2a, b). Briefly, germ cells, Leydig cells, Sertoli cells, peritubular cells, macrophages, and endothelial cells could be clearly distinguished. The germ cell cluster shows not only the expression of pan-germ cell marker *VASA/DDX4*, but also meiotic (*MEIOB*, *SYCP3*) and spermatid (*PRM1*) marker genes, indicating full spermatogenic process in the tissue analysed. In order to understand whether these germ cells show normal transcriptome profiles, we compared them to a well-characterized published dataset [25]. Germ cells from both datasets were subset together and re-clustered. Specific germ cell types were assigned by using known differentiation markers (Fig. 2c, d). The 39 germ cells obtained from the KS patient clustered together with the “normal” germ cell dataset (Fig. 2c). As the cell numbers obtained from KS testicular biopsies were too low to perform single-cell RNA-seq and DNA methylation analyses on the same samples, the former analysis was limited to one Klinefelter sample. Consequently, no differential expression analysis was performed to identify potentially existing subtle differences. However, based on similar clustering, no gross changes in germ cell transcriptome appear to be present.

### DNA methylation at single-allele resolution in adult human germ cells from normal samples

**Fig. 3:**
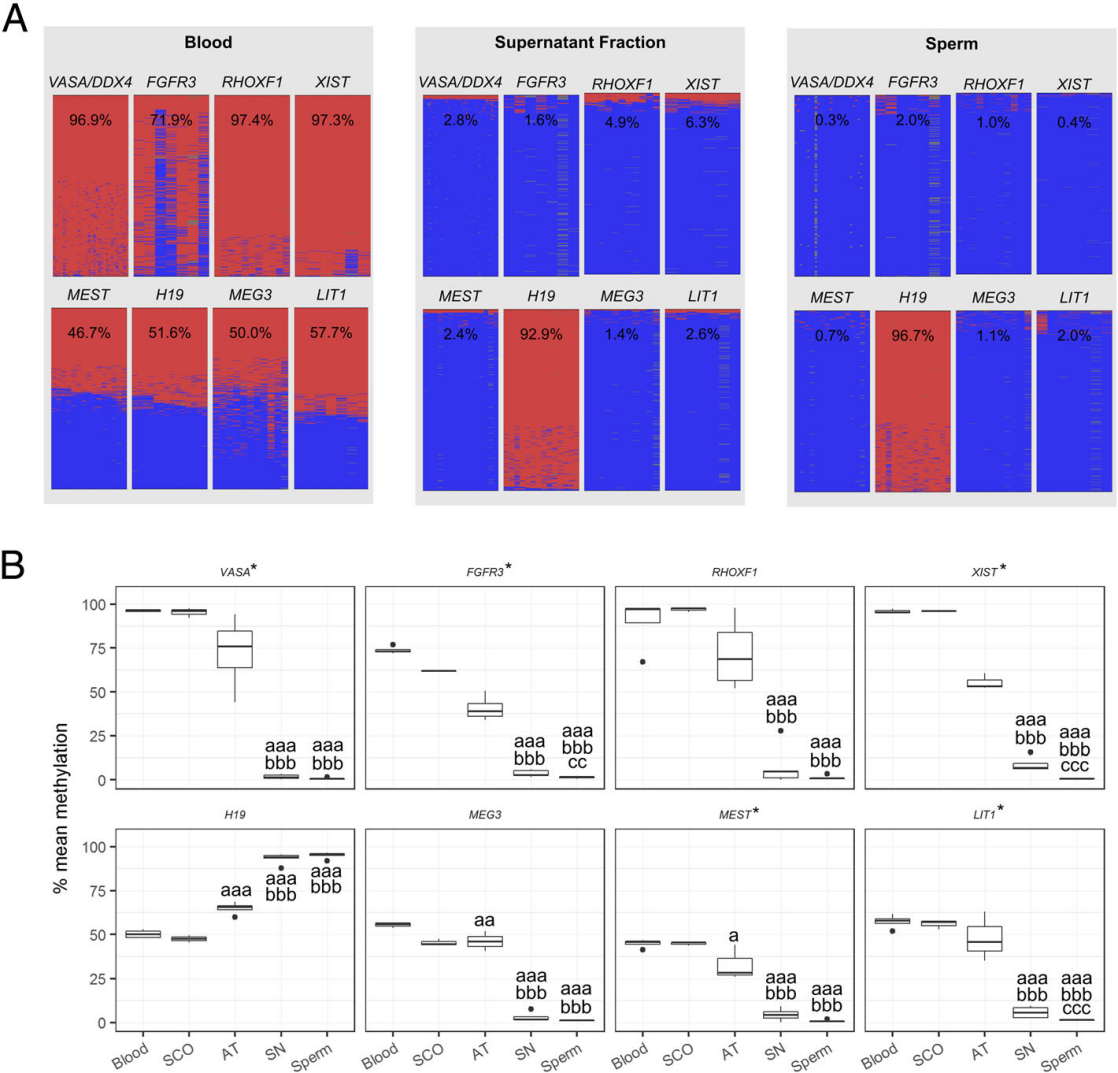
Single-allele resolution analysis of DNA methylation levels in undifferentiated germ cells (supernatant, SN) and sperm. Blood, Sertoli cell-only (SCO) samples, and testicular somatic cells (AT) are shown as somatic controls. **a** Exemplary DNA methylation plots for a blood sample, a supernatant fraction, and a sperm sample. Each column represents a CpG position while each line corresponds to an individual sequencing read. Methylated positions are denoted by the colour red and unmethylated by the colour blue. **b** Box plots displaying the DNA methylation levels for the included samples. An asterisk denotes that the methylation values were log-transformed in order to approach normality before statistical analysis (untransformed data is displayed for ease of interpretation). Statistically significant differences in average DNA methylation levels are denoted by letters: a—different from blood, b—different from AT fraction, c—different from SN fraction. *p* values are denoted by the number of letters, e.g. a—*p* < 0.05, aa—*p* < 0.005, aaa—*p* < 0.001

In order to evaluate the DNA methylation patterns of germ cells in normal samples, we performed DBS at single-allele resolution (Fig. 3). To gauge the purity of the cultures from the normal group, we included the somatic-rich AT fraction and blood as controls. We analysed three germ cell marker genes (*FGFR3*, *RHOXF1*, *VASA /DDX4*) as well as *XIST*, which was shown to be unmethylated in the male germline. In addition, two maternally—*MEST*:alt-TSS-DMR (*MEST*), KCNQ1OT1:TSS-DMR (*LIT1*)—and two paternally imprinted genes—*H19/IGF2*:IG-DMR (*H19*), *MEG3*:TSS-DMR (*MEG3*) (nomenclature of imprinted genes as published in [26])—were analysed. All germ cell-enriched SN fractions presenting *VASA/DDX4* methylation higher than 4% were excluded from the analysis due significant presence of somatic cells in the cultures. Representative methylation plots are shown for blood, supernatant fraction, and sperm (Fig. 3a). In blood and testicular somatic cells (Sertoli cell-only samples, SCO), the four imprinted genes showed similar amounts of methylated and unmethylated alleles while the germ cell markers and *XIST* are fully methylated (Fig. 3a, b). The germ cell-enriched SN fraction shows DNA methylation patterns for all studied regions which are highly comparable to those found for sperm (Fig. 3a, b).

### DNA methylation profile at single-allele resolution of germ cell marker genes in Klinefelter samples

**Fig. 4:**
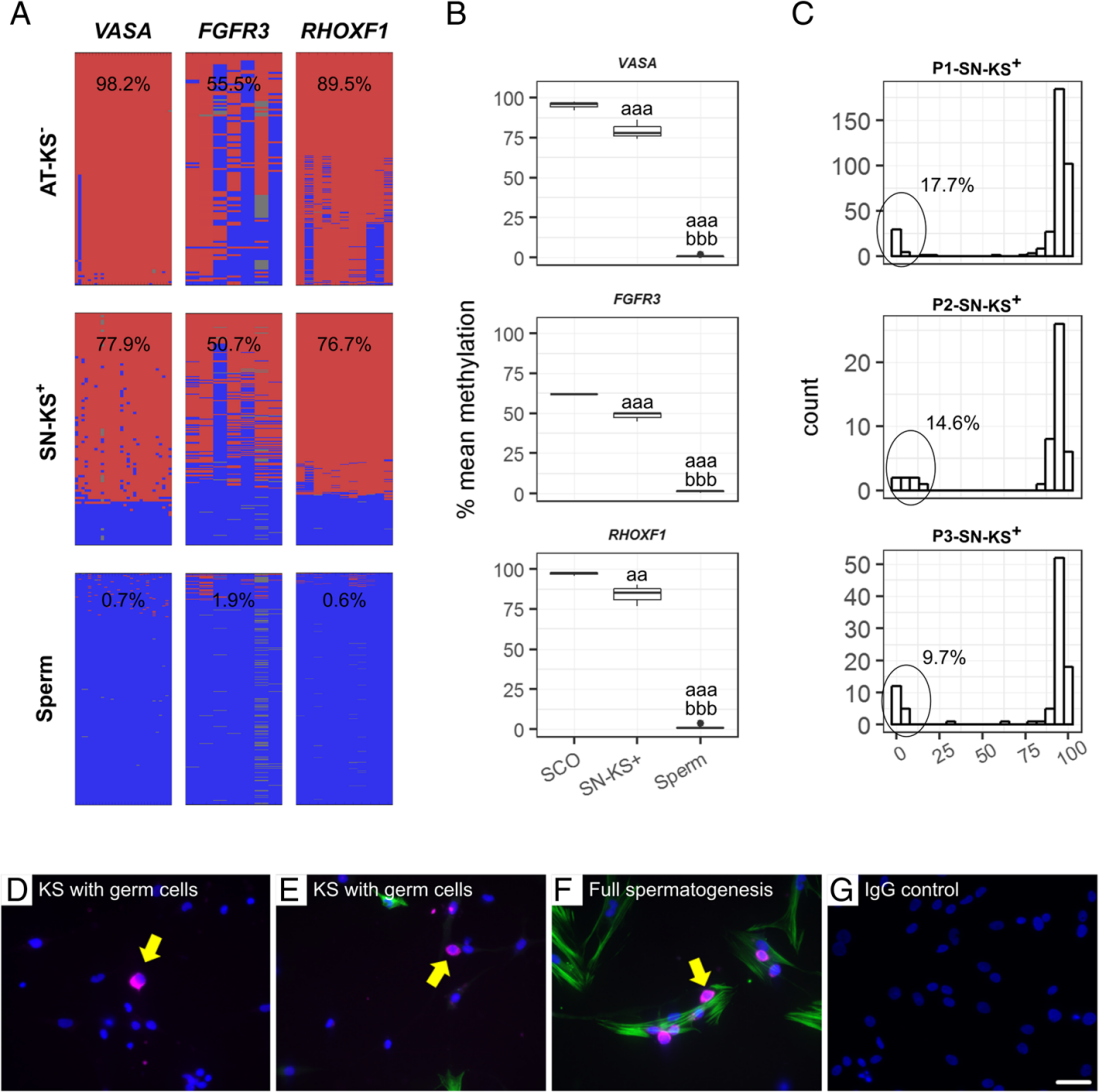
Germ cells in cultures from Klinefelter (KS) men detected at protein and DNA methylation levels. **a** Representative plots of DNA methylation patterns for the three germ cell marker genes in a somatic-cell attached fraction from a KS patient without germ cells (AT-KS(−)), germ cell-containing supernatant fraction from a KS patient with germ cells (SN-KS(+)), and sperm as a germ cell control. Each column represents a CpG position while each line corresponds to an individual sequencing read. Methylated positions are denoted in red and unmethylated in blue. **b** Mean DNA methylation values of each germ cell marker gene in SN-KS+ (*n* = 3) were compared to a pure testicular somatic control with Sertoli cell-only phenotype (SCO, *n* = 3) and sperm as a pure germ cell control (*n* = 5). The presence of germ cells in the KS sample can be observed as a shift downwards in the DNA methylation levels compared to SCO. Statistically significant differences in average DNA methylation levels are denoted by letters: a—different from SCO, b—different from SN-KS. *p* values are denoted by the number of letters (e.g. a—*p* < 0.05, aa—*p* < 0.005, aaa—*p* < 0.001). **c** Histograms showing the read distribution of *VASA/DDX4* DNA methylation in the three SN-KS+ samples. Based on the proportion of unmethylated reads, it is possible to estimate the amount of germ cells contained within the analysed sample (displayed in each individual panel). **d–g** Micrographs showing immunofluorescence staining for VASA/DDX4 (magenta), α-smooth muscle actin (αSMA, green), and DAPI (blue) of testicular cells in culture. As a comparison, a culture from a man with full spermatogenesis **f** is shown. The negative control **g** showed no immunological staining. Scale bar = 50 μm

In KS samples with germ cells (SN-KS+), fully methylated reads typical for somatic cells were observed but also a fraction of fully unmethylated reads identical to the patterns obtained for sperm and SN fractions from men with full spermatogenesis for the three germ cell markers examined (Fig. 4a). As can be observed (Fig. 4b), SN fractions from KS men containing germ cells had statistically lower DNA methylation in all four germ cell marker genes analysed than the SCO samples (used as somatic-only control). This was due to the presence of a population of unmethylated reads originating in germ cells in the KS samples. By calculating the proportion of unmethylated reads derived from each KS sample, we could estimate the percentage of germ cells in each culture (Fig. 4c). Moreover, by using immunofluorescence, the presence of germ cells in KS testicular cultures was confirmed at the protein level, as shown by VASA/DDX4 expression (Fig. 4d–g).

### DNA methylation profile at single-allele resolution of imprinted genes in Klinefelter samples

**Fig. 5:**
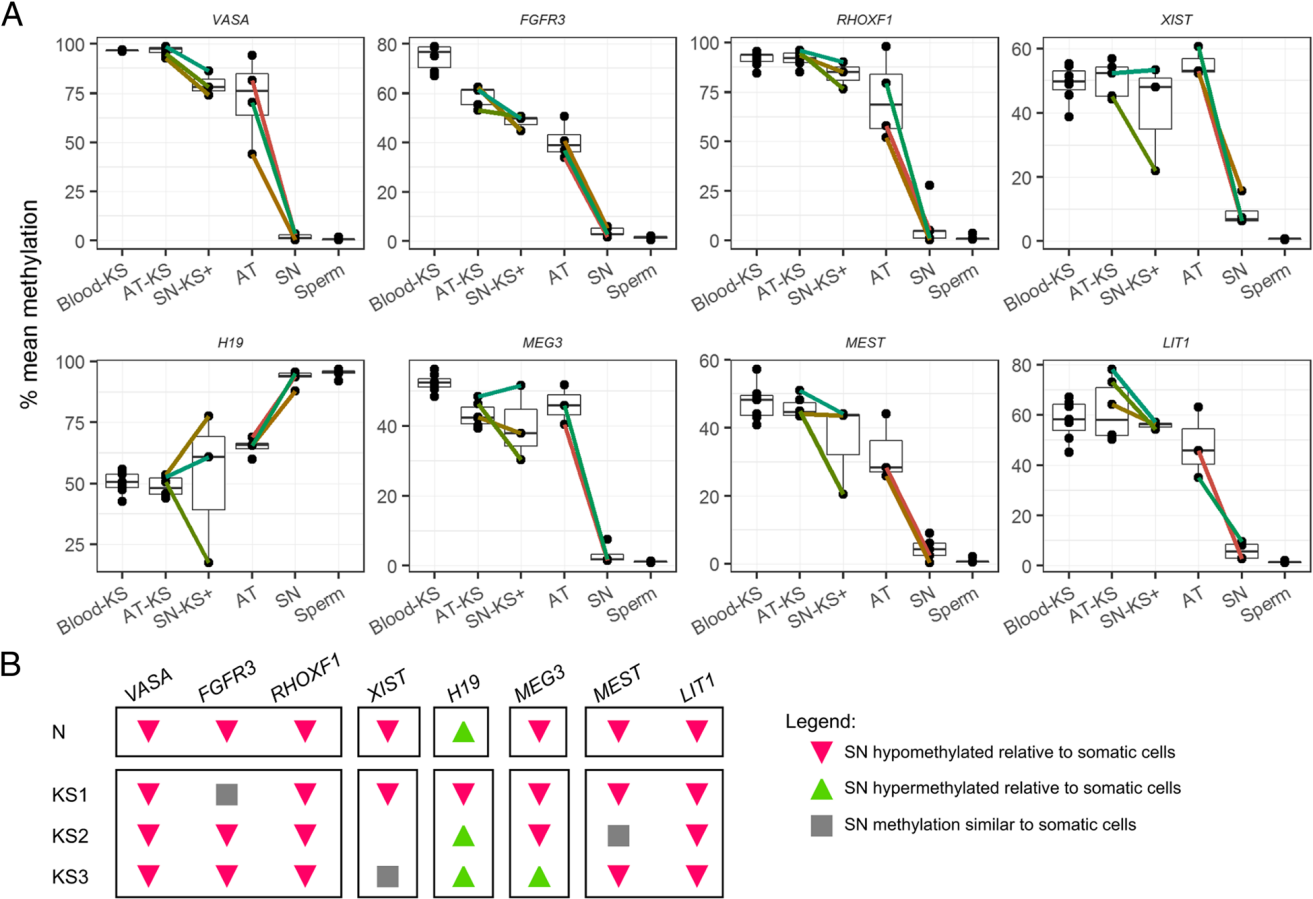
DNA methylation in germ (SN) and somatic cell (AT) fractions in normal and Klinefelter patients. **a** Samples obtained from the same individual are connected by individually coloured lines. **b** Summary of the pattern of DNA differences between somatic and germ cells. Only a representative pattern is shown for normal samples due to the homogeneity of the patterns. Blood-KS blood from Klinefelter syndrome patients, AT-KS somatic cell fraction from Klinefelter samples, SN-KS+ germ cell enriched fraction from Klinefelter samples, AT somatic fraction from normal man, SN germ cell fraction from normal man

We next assessed the methylation status of imprinted genes and *XIST* (Fig. 5) by comparing germ cell (SN) and somatic fractions (AT) of both Klinefelter and normal men. The enrichment in germ cells in the supernatant fraction from Klinefelter men with focal spermatogenesis could be observed (similarly to normal controls) as a reduction in DNA methylation levels of germ cell markers, when compared with the somatic fractions. Akin to those, a decrease (for maternally imprinted genes and *XIST*) or increase (for paternally imprinted genes) in DNA methylation for each germ cell fraction of Klinefelter samples, compared to the respective somatic fraction, was expected. This shift should be proportional to the relative amount of germ cells present in the fraction and should be constant for all genes analysed, provided that no aberrations in DNA methylation are present in Klinefelter germ cells. Regarding the apparent difference between the methylation levels of AT fractions in normal and Klinefelter samples, this could be due to the residual presence of germ cells in the former. For normal samples, we could observe a proportional decrease in the DNA methylation level germ cell markers (Fig. 5, upper panel) as well as for *MEG3*, *MEST*, *LIT1*, and *XIST* and an increase in the levels of *H19* in testicular germ cells compared to the corresponding somatic cell fractions.

In contrast, this expected pattern of methylation change could not be observed for KS samples. All three Klinefelter testicular samples analysed show divergence from the expected pattern of increased and decreased methylation in the imprinted genes analysed. As clearly seen in Fig. 5, for one patient, *H19* methylation levels decreased in the germ cell fraction instead on increasing; for a second patient, the levels of *MEG3* increased instead of decreasing; and for the third, there was no change in the methylation levels of *MEST* between somatic and germ cell-enriched fraction. Along with an unexpected decrease in DNA methylation of *H19*, the same germ cell fraction of one patient also displayed an exaggerated decrease in *XIST* methylation. Solely for *LIT1* did all three KS+ samples show the expected pattern.

## Discussion

The aim of this study was to investigate the DNA methylation patterns and the transcriptome of germ cells from Klinefelter patients and compare them to germ cells from men with complete spermatogenesis. For this purpose, we have used DNA methylation analysis at single-allele resolution and single-cell RNA-Seq. Sperm isolated from the Klinefelter testicular tissue is routinely used for ART in clinical practice to help these men become biological fathers. Yet, surprisingly, little is known about the molecular features and programming of germ cells in this common type of aneuploidy. This fact was highlighted in 2016 during the International Workshop on Klinefelter Syndrome, in which it became clear that there is a gap in knowledge of the molecular features of Klinefelter men and specifically Klinefelter gametes [24]. As spermatozoa from Klinefelter men are rare and difficult to obtain, we have instead focused on studying testicular germ cells obtained from biopsies presenting with focal spermatogenesis.

A question we first addressed was whether testicular germ cells have similar DNA methylation patterns as sperm. An early report seemed to indicate that imprints were only completely established during the course of spermatogenesis [27]. However, more recent publications indicated that DNA methylation levels of selected imprinted genes were comparable between spermatogonia and sperm [28]. Also recently, bulk analysis at genome-wide level has shown that human spermatogonia sorted for marker SSEA4 shows DNA methylation patterns which are not overall different from sperm in regards to pluripotency, meiotic, and imprinted genes [29]. Importantly, we have also shown that in a non-human primate model, DNA methylation of selected imprinted and germ cell marker genes is established during pubertal development and is maintained at similar levels between spermatogonia and sperm. Moreover, these methylation patterns remained stable throughout 3 weeks of cell culture [30], rendering the approach of differential plating feasible to further assess the methylation status of human germ cells. We could confirm that in adult testicular germ cells, the methylation patterns of the studied imprinted (*H19*, *MEG3*, *MEST*, *LIT1*) and germ cell marker genes (*FGFR3*, *DDX4*, *RHOXF1*), and *XIST* are comparable to those of sperm. Moreover, there was very little inter- and intra-individual variation in the five samples analysed. Therefore, the methylation of these key regions is already fully in place in the earlier stages of spermatogenesis and is not subject to methylation changes during spermatogenesis. Nevertheless, this is not to say that other regions might be subjected to methylation and demethylation dynamics during the process of spermatogenesis, as shown previously in rodent studies [31, 32]. The surprisingly homogeneous patterns found in this study highlight the robustness of DNA methylation profiles in the male germline and, importantly, shows that these regions are not affected by the cell culture strategy used to obtain the germ cell and somatic fractions studied.

In addition to germ cell markers and imprinted genes, we also analysed the DNA methylation pattern of *XIST*. In women, one copy of *XIST* is fully methylated while the other remains unmethylated, resulting in a degree of methylation around 50% for somatic tissues. Similarly, in Klinefelter men with XXY karyotype, the same DNA methylation values were found [16]. In contrast to somatic cell types, there are indications that *XIST* might be subjected to a different regulation in the germline [33]. When we evaluated the DNA methylation of *XIST* in sperm, we could verify that the promoter for this gene is fully unmethylated. Likewise, in testicular germ cell fractions, we found a similar pattern of DNA methylation, in contrast to testicular somatic cells where it remains fully methylated. These results demonstrate that *XIST* remains stably unmethylated in the adult male germline, although its function in the context of spermatogenesis remains to be elucidated.

To enrich germ cells of KS men with focal spermatogenesis for further analysis, we employed a cell culture approach. We were able to demonstrate the presence of germ cells in these fractions not only by the expression of the germ cell marker protein VASA/DDX4, but also by single-cell RNA-Seq and DNA methylation analysis. Using single-cell RNA-Seq, we were able to obtain the transcriptional profiles of multiple cell types present in the testis. In the sample that was analysed, we could detect 39 cells displaying gene expression patterns consistent with a germ cell origin. We compared these cells with those originating from men with full spermatogenesis from a previously published study [25]. Unsupervised clustering of the germ cells revealed that germ cells derived from the Klinefelter patient cluster together with those obtained from fertile men. This appears to indicate that there are no significant changes in the transcriptome of these few cells that remain functional in the testes of KS patients. The analysis of the DNA methylation patterns of germ cell markers showed similar findings. We were able to demonstrate the presence of a germ cell population with highly homogeneous and normal DNA methylation patterns for *FGFR3*, *VASA/DDX4*, and *RHOXF1*, the latter being an X-linked gene (Fig. 4).

A number of studies have described changes in DNA methylation in the sperm of infertile men [34]. These studies have ranged from the analysis of global DNA methylation [35] and repetitive regions [36], to imprinted [37] and germ cell-specific genes [38, 39]. A recent meta-analysis has confirmed this association between male infertility and aberrant sperm DNA methylation, in particular for imprinted regions *H19*, *MEST*, and *SNRPN* [21].

However, it is unknown whether the sperm of KS men might be similarly affected by changes in DNA methylation. This is because the number of sperm obtained from these men (usually by micro-TESE) is too small to allow DNA methylation analysis to be performed. In this study, we were able to analyse DNA methylation, at the single-allele resolution, in the germline of both normal and Klinefelter men by studying the germ cell-enriched fraction obtained after differential platting. In normal men, this allows the collection of a highly pure germ cell fraction that can be used for methylation and transcriptional analysis without interference of other cell types. However, the number of germ cells obtained from the testes of men with this aneuploidy is too small and only enrichment is possible, without complete separation from somatic cells. Nevertheless, we were able to detect a portion of reads compatible with the presence of normal germ cells based on the DNA methylation of germ cell markers.

For *XIST*, testicular somatic cells from KS patients showed a pattern of DNA methylation that is similar to haematopoietic lineages (i.e. around 50%), reflecting the inactivation of the supernumerary X chromosome. The enrichment in germ cells in the supernatant fraction from Klinefelter men with focal spermatogenesis could be observed (similarly to germ cell markers) as a reduction in the DNA methylation levels, when compared with the somatic fractions. Akin to *XIST*, a decrease (for maternally imprinted genes and *XIST*) or increase (for paternally imprinted genes) in DNA methylation for each germ cell fraction, compared to the respective purely somatic fraction, was expected. That was however not always the case, as there were serious deviations in the expected trends (Fig. 5). Our data indicates that Klinefelter germ cells are prone to epimutations in imprinted genes, and considering we found these aberrations in three out of three samples tested, these epimutations might be more prevalent in this patient group than in euploid infertile men. Nevertheless, this hypothesis can only be tested by using single-cell DNA methylation methods, which does not yet provide sufficient coverage for this purpose. Moreover, the Klinefelter germ cells analysed did not include sperm, which would be ultimately necessary in order to understand if these abnormal germ cells at all produce gametes that would be available for ART procedures. Nevertheless, KS is known to affect the methylation status of a large number of loci, both autosomal as well as sex chromosome-linked, in haematopoietic lineages [19], therefore it is not far-fetched that germ cell lineages are also affected by this epigenetic instability.

## Conclusions

DNA methylation patterns of imprinted genes as well as germ cell marker genes are already in place and remain stable throughout spermatogenesis in normal samples. Interestingly, *XIST* shows a pattern of regulation that distinguishes the germline from somatic tissues. This gene, which is regularly fully methylated in normal males, shows a completely unmethylated status in undifferentiated germ cells as well as sperm. Individual Klinefelter men showed the presence of a population of germ cells with normal transcription as well as DNA methylation patterns for germ cell markers. In stark contrast, imprinted genes did not show the expected patterns of DNA methylation, indicating that the germline from these men might present imprinting aberrations.

## Methods

### Selection of testicular biopsies and ethical approval

EDTA-blood and sperm samples (Institutional Review Board approval: 4INie) were obtained from patients presenting at the Department of Clinical and Surgical Andrology at the University Hospital in Münster, Germany.

For sperm samples, semen analyses were performed in accordance with the guidelines of the World Health Organization [40], including evaluation of sperm count, motility, and morphology. Each sperm sample was collected following pelleted swim-up procedure to isolate the motile sperm, and based on this, normozoospermic samples were selected [37].

In patients presenting with obstructive azoospermia or non-obstructive hypergonadotropic azoospermia (including Klinefelter patients and SCO patients), one additional testicular biopsy was obtained during routine surgical procedure for therapeutic testicular sperm extraction (TESE) and histological analysis, following ethical approval and written informed consent (Ethics Committee of the Medical Faculty of Münster and the State Medical Board no. 2008-090-f-S). Testicular tissues were selected from 444 consecutively obtained biopsies over a 2-year period. Suitable samples were assigned to the categories normal (obstructive azoospermia and full spermatogenesis), SCO syndrome, and KS. Testicular tissues were immediately placed in chilled MEMα medium (Life Technologies GmbH, Gibco, Darmstadt, Germany) for transfer to the cell culture laboratory.

Additional file 2: Table S1 provides an overview of all samples included in this study and the analyses to which they were subjected.

### Histological and immunohistochemical analysis of human testicular tissue samples

Testicular tissues were fixed in Bouin’s solution overnight and subsequently washed and stored in 70% (v/v) ethanol. For routine histological evaluation, tissues were paraffin-embedded and sectioned at 5 μm for periodic acid-Schiff/haematoxylin staining [41]. Sections from two independent biopsies were assessed and the most advanced germ cell type was determined for all seminiferous tubules. The percentage of tubules with tubular ghosts and Sertoli cell-only (SCO) phenotype was also determined.

### Endocrine and histological parameters of selected normal and Klinefelter syndrome patient groups

Tissues were considered normal provided that elongated spermatids were present in at least 60% of seminiferous tubules in the histological analysis and sperm was detectable in all TESE samples (Table 1). In contrast, samples were considered as SCO only if there were no germ cells in seminiferous tubules and no sperm in any of the TESE samples (Table 1). Due to the persistence of germ cells, only in focal areas of KS testes, the histological analysis, TESE results, and the biopsy available for research yielded inconsistent results regarding the presence of germ cells. Following the confirmation of germ cell presence by microscopical evaluation in four out of seven samples, we have labelled the former KS+.

**Table 1:** Parameters of the patient samples included

Patients	Karyotype	Total testis volume (ml)	Histological parameters					Sperm (mTESE)
			% SPT	% SPC	% SPG	% SCO	% TS	
Normal (*n* = 5)	Not determined	20.7 (± 11.2)	66.0 (± 13.4)	22.2 (± 8.9)	1.6 (± 1.4)	0.2 (± 0.4)	1.4 (± 1.0)	Yes
SCO (*n* = 3)	46,XY	8.7 (± 3.8)	0	0	0	99.0 (± 1.0)	1.0 (± 1.0)	No
KS+ (*n* = 4)	1: 48,XXYY 2–4: 47,XXY	7.8 (± 5.7)	0.3 (0.5)	1,75 (± 2.9)	0	73,3 (± 25.6)	24.3 (± 26.9)	1, yes; 2–4, no
KS− (*n* = 3)	1–3: 47,XXY	2.0 (± 0)	0	0	0	19.3 (± 11.2)	80.7 (± 11.2)	1 no; 2, yes; 3, yes

Mean values of the testicular volumes per patient group are given as well as the percentage of tubules showing the most advanced stage of germ cell differentiation. Finally, it is indicated whether mTESE samples contained sperm

*KS* Klinefelter syndrome, + with testicular germ cells, − without testicular germ cells, *mTESE* microsurgical testicular sperm extraction, *Normal* testicular tissues with qualitatively normal spermatogenesis, *SCO* Sertoli cell-only syndrome, *SPC* spermatocyte, *SPG* spermatogonia, *SPT* spermatid, *TS* tubular shadows

Hormone measurements for gonadotropins and testosterone were performed in the frame of the clinical routine analysis, and methods were as previously described [42]. Endocrinologically, the patient group that was considered ‘normal’ was characterized by FSH, LH, and testosterone levels within the normal range (Additional file 2: Table S2). The SCO patient group had significantly elevated FSH levels (*p* < 0.05). In the KS group, there was elevated FSH and LH, which importantly could not predict the presence or absence of germ cells.

### Digestion of human testicular tissues and short-term culture of testicular tissue fractions

To achieve separation of testicular somatic cells from germ cells, a two-step differential plating approach was employed following enzymatic digestion of testicular tissues as previously published [43]. Cells were plated onto uncoated 6-cm culture dishes in MEMα medium (Life Technologies GmbH, Gibco, Darmstadt, Germany) supplemented with 10% FCS (foetal calf serum) and 1% Pen/Strep in an atmosphere of 35 °C and 5% CO^2^. The supernatant cell fraction was separated from the attached cells after overnight culture. Based on previous studies [43], enrichment of spermatogonia was highest following 4–5 days of in vitro culture, as the somatic cells were morphologically distinguishable and firmly attached to the culture plate.

Differential plating of testicular cell suspensions with normal spermatogenesis yielded a germ cell-enriched (SN; Additional file 1: Figure S2A) fraction and a somatic cell-rich (AT; Additional file 1: Figure S2B) fraction. Biopsies from patients with an SCO phenotype yielded only an AT fraction (Additional file 1: Figure S2C). In KS samples with germ cells (KS+; Additional file 1: Figure S1D), both SN and AT fraction (KS+; Additional file 1: Figure S1E) were obtained and the presence of germ cell cluster was microscopically confirmed. In the KS− group, only AT fractions could be recovered (Additional file 1: Figure S2F).

### RNA expression analysis of germ cell marker genes

RNA was isolated from cell pellets containing 20,000–150,000 cells employing the miRNeasy micro Kit (Qiagen) following the manufacturer’s instructions and including DNAse digestion. For reverse transcription, the iScript cDNA synthesis kit (BioRAD) and 100 ng RNA were used as starting material. Primer sequences for *DDX4* and *FGFR3* [42] and for *RHOXF1* and *RHOXF2* [44] were previously published (Additional file 2: Table S3). SYBR Green-based quantitative real-time PCR analyses were run on a StepOnePlus^TM^ machine and analysed using the Step One^TM^ software. Following normalization of data to the reference gene *GAPDH*, data were plotted as 2^−ΔCt^ values.

### Single-cell RNA sequencing of a Klinefelter sample

A testicular biopsy from a Klinefelter patient was subjected to single-cell RNA-Seq following enzymatic digestion [43] and passage through a 70-μm strainer (Miltenyi Biotec). Specifically, for library preparation, a single-cell suspension with 500 cells/μl was used for library preparation according to the instructions of the kit (10x Genomics Chromium Single Cell Reagent Kit v2) with the exception of using Beckman Coulter Agencourt AMPure XP for library purification. The cell suspension was loaded onto a Chromium Single Cell A Chip using a 10x Genomics Chromium controller. Twelve thousand cells were loaded onto the chip and about 6000 embedded single cells were obtained after processing. cDNA amplification and library quality were controlled and quantified using the Lab901 TapeStation system (Agilent).

The libraries were sequenced twice. First, shallow sequencing was performed on a NextSeq-550 sequencer (Illumina) as a quality control measure (data not shown). The final, deep sequencing was performed on a NovaSeq 6000 sequencer (Illumina), using 2 × 150 bp paired-end sequencing. Shallow sequencing was performed in the Core Facility for High Throughput Genetics and Genomics of the Medical Faculty of the University of Münster. Deep sequencing was performed by MicroAnaly Gene Technologies Co., Ltd (Shanghai, China).

Demultiplexed sequencing reads were mapped to the human genome (GRCh38) using 10x Cell Ranger (v2.1.1). Primary data analysis and quality control were also performed using the Cell Ranger pipeline. Filtered matrixes were obtained and loaded onto R for further analysis using Seurat (v2.3.0) [45]. Cell types were identified based on known marker expression [25]. Publicly available single-cell transcriptome data [25] was used as a normal spermatogenesis control. Briefly, filtered matrixes were combined with KS single-cell data using Seurat. The combined dataset was filtered and normalized, and then clustering was performed based on known markers [25]. Germ cells were subset and reanalysed separately.

### Deep bisulfite sequencing analyses

DNA was purified from cultured cell fractions, swim-up sperm (prepared by pellet swim-up), or blood. For cultured cell fractions and swim-up sperm, the MasterPure DNA purification kit (Epicentre Biotechnologies) was used, according to the protocol provided by the manufacturer for use with cell samples and a modified protocol [37], respectively. DNA was isolated from EDTA-blood using the FlexiGene DNA kit (Qiagen) according to the instructions supplied by the manufacturer. DNA concentration was measured by spectrophotometry (NanoDrop ND-1000, Peqlab, Erlangen, Germany) and the samples were stored at − 20 °C until further use. Two hundred nanograms of DNA were converted using the EZ DNA methylation Gold kit (Zymo). Locus-specific libraries for deep bisulfite sequencing (DBS) were prepared by two rounds of PCR amplification as previously described [37, 46]. In the first round, locus-specific tagged primers (Additional file 2: Table S4) were used. In the second round, sample-specific barcode sequences (MID, multiplex identifiers) and universal linker tags (454 adaptor sequences) were added (95 °C, 15 min; 35 × (95 °C, 30 s; 72 °C, 1 min); 72 °C, 10 min). Sample preparation and sequencing were performed using the Roche/454 GS Junior platform, as described elsewhere [35, 44]. The read yield was increased by special filter settings [47]. Analysis of DNA methylation was done using Amplikyzer2.0 [48].

### Inclusion criteria for germ cell fractions based on sample purity

We initially analysed eight SN fractions from patients with qualitatively normal spermatogenesis by DBS. As a quality control measure, we used the DNA methylation levels of *VASA/DDX4* to ensure purity of the germ cell fraction. Three samples with over 4% *VASA/DDX4* DNA methylation were considered as containing somatic cells and were therefore excluded from subsequent analysis. Ultimately, for euploid men, five SN, four AT, three SCO, five blood, and six sperm samples were included in the analysis. For KS samples, seven blood, three SN, and three AT from men with germ cells and three AT from samples without germ cells were included.

### Immunohistochemical and immunofluorescence staining of testicular cells

For immunohistochemical analyses, Bouin’s fixed and paraffin-embedded testicular tissues were used and sectioned at 3 μm. Primary and secondary antibodies were DDX4 (VASA; AF2030, R&D Systems; 1:100) and chicken anti-goat-biotin (SC2984, Santa Cruz; 1:100), respectively. The protocol was as previously published [49] using isotype control and omission of primary antibody as negative controls. The immunofluorescence staining was performed as previously described [42]. Briefly, the digested testicular cells were plated in eight-well chamber slides. After 3 days of culture, the cells were fixed with 4% PFA in phosphate-buffered saline (PBS) for 30 min at room temperature and permeabilized with 0.5% Triton X-100 (Sigma-Aldrich, 93443). The nonspecific background was reduced by incubating the cells with 1% bovine serum albumin (BSA; Sigma-Aldrich, A9647) and 20% Donkey serum (Jackson Laboratories Immuno Research). Subsequently, the cells were incubated overnight at 4 °C with anti-DDX4 antibody (Abcam, AB13840; 1:1000) and anti-αSMA antibody (Sigma-Aldrich, A2547; 1:1000). As a negative control, one well per sample was incubated with rabbit and mouse IgG (1:1000). After washing, the cells were incubated for 1 h with donkey anti-rabbit CyTM3 (Jackson Laboratories Immuno Research, 775-546-150; 1:400) and donkey anti-mouse 488 (Jackson Laboratories Immuno Research, 711-166-152; 1:400). Slides were mounted with Vectashield Mounting Medium with 4,6-diamidino-2-phenylindole as nuclear counterstain (Vector Laboratories, Inc., Burlingame, CA, USA).

### Statistical analyses

Results are shown as mean ± SEM. All data were checked for normality of distribution and, if necessary, log-transformed to approach normality before comparing different means by analysis of variance (ANOVA), followed by pairwise *t* tests (with Holm’s correction for multiple testing). All statistical tests and graphs were done using R [50] .

## Additional files

Additional file 1: **Figure S1.** Relative gene expression data from human germ cell and somatic cell fractions compared to the initial testicular cell population. **Figure S2.** Phase contrast micrographic images of human testicular cell cultures. (DOCX 1491 kb)

Additional file 2: **Table S1.** Overview of patient samples included in this study and analyses to which they were subjected. **Table S2.** Endocrine parameters of the patient samples. **Table S3.** Gene names and primer sequences for transcript expression (using quantitative PCR) of selected germ cell marker genes. **Table S4.** Gene names and primer sequences for DNA methylation analysis (by deep bisulfite sequencing) of imprinted genes, selected germ cell markers, and *XIST*. (DOCX 22 kb)

## Data Availability

All single-cell sequencing data are publicly available from the GEO database through accession number GSE130151.

## References

[1] Tüttelmann F, Gromoll J (2010). Novel genetic aspects of Klinefelter's syndrome. Mol Hum Reprod.

[2] Frühmesser A, Kotzot D (2011). Chromosomal variants in klinefelter syndrome. Sex Dev.

[3] Lanfranco F, Kamischke A, Zitzmann M, Nieschlag E (2004). Klinefelter's syndrome. Lancet.

[4] Ferguson-Smith MA (1959). The prepubertal testicular lesion in chromatin-positive Klinefelter's syndrome (primary micro-orchidism) as seen in mentally handicapped children. Lancet.

[5] Wikström AM, Raivio T, Hadziselimovic F, Wikström S, Tuuri T, Dunkel L (2004). Klinefelter syndrome in adolescence: onset of puberty is associated with accelerated germ cell depletion. J Clin Endocrinol Metab.

[6] Rohayem J, Fricke R, Czeloth K, Mallidis C, Wistuba J, Krallmann C (2015). Age and markers of Leydig cell function, but not of Sertoli cell function predict the success of sperm retrieval in adolescents and adults with Klinefelter's syndrome. Andrology.

[7] Vialard F, Bailly M, Bouazzi H, Albert M, Pont JC, Mendes V (2012). The high frequency of sperm aneuploidy in klinefelter patients and in nonobstructive azoospermia is due to meiotic errors in euploid spermatocytes. J Androl.

[8] Greco E, Scarselli F, Minasi MG, Casciani V, Zavaglia D, Dente D (2013). Birth of 16 healthy children after ICSI in cases of nonmosaic Klinefelter syndrome. Hum Reprod.

[9] Madureira C, Cunha M, Sousa M, Neto AP, Pinho MJ, Viana P (2014). Treatment by testicular sperm extraction and intracytoplasmic sperm injection of 65 azoospermic patients with non-mosaic Klinefelter syndrome with birth of 17 healthy children. Andrology.

[10] Sciurano RB, Luna Hisano CV, Rahn MI, Brugo Olmedo S, Rey Valzacchi G, Coco R, Solari AJ (2009). Focal spermatogenesis originates in euploid germ cells in classical Klinefelter patients. Hum Reprod.

[11] Zitzmann M, Bongers R, Werler S, Bogdanova N, Wistuba J, Kliesch S (2015). Gene expression patterns in relation to the clinical phenotype in Klinefelter syndrome. J Clin Endocrinol Metab.

[12] Penny GD, Kay GF, Sheardown SA, Rastan S, Brockdorff N (1996). Requirement for Xist in X chromosome inactivation. Nature.

[13] Clemson CM, McNeil JA, Willard HF, Lawrence JB (1996). XIST RNA paints the inactive X chromosome at interphase: evidence for a novel RNA involved in nuclear/chromosome structure. J Cell Biol.

[14] Tinker AV, Brown CJ (1998). Induction of XIST expression from the human active X chromosome in mouse/human somatic cell hybrids by DNA demethylation. Nucleic Acids Res.

[15] Zuccotti M, Monk M (1995). Methylation of the mouse Xist gene in sperm and eggs correlates with imprinted Xist expression and paternal X-inactivation. Nat Genet.

[16] Poplinski A, Wieacker P, Kliesch S, Gromoll J (2010). Severe XIST hypomethylation clearly distinguishes (SRY+) 46,XX-maleness from Klinefelter syndrome. Eur J Endocrinol.

[17] Winge SB, Dalgaard MD, Belling KG, Jensen JM, Nielsen JE, Aksglaede L, et al. Transcriptome analysis of the adult human Klinefelter testis and cellularity-matched controls reveals disturbed differentiation of Sertoli- and Leydig cells. Cell Death Dis. 9:586. 10.1038/s41419-018-0671-1. 10.1038/s41419-018-0671-1 PMC5964117 29789566

[18] Wan ES, Qiu W, Morrow J, Beaty TH, Hetmanski J, Make BJ (2015). Genome-wide site-specific differential methylation in the blood of individuals with Klinefelter syndrome. Mol Reprod Dev.

[19] Sharma A, Jamil MA, Nuesgen N, Schreiner F, Priebe L, Hoffmann P (2015). DNA methylation signature in peripheral blood reveals distinct characteristics of human X chromosome numerical aberrations. Clin Epigenetics.

[20] Skakkebæk A, Nielsen MM, Trolle C, Vang S, Hornshøj H, Hedegaard J (2018). DNA hypermethylation and differential gene expression associated with Klinefelter syndrome. Sci Rep.

[21] Santi D, de VS, Magnani E, Spaggiari G (2017). Impairment of sperm DNA methylation in male infertility: a meta-analytic study. Andrology.

[22] Matzuk MM, Lamb DJ (2008). The biology of infertility: research advances and clinical challenges. Nat Med.

[23] Fauser BCJM, Devroey P, Diedrich K, Balaban B, Bonduelle M, de D-v WHA (2014). Health outcomes of children born after IVF/ICSI: a review of current expert opinion and literature. Reprod BioMed Online.

[24] Nieschlag E, Ferlin A, Gravholt CH, Gromoll J, Köhler B, Lejeune H (2016). The Klinefelter syndrome: current management and research challenges. Andrology.

[25] Hermann BP, Cheng K, Singh A, La Roa-De Cruz L, Mutoji KN, Chen I-C (2018). The Mammalian Spermatogenesis Single-Cell Transcriptome, from Spermatogonial Stem Cells to Spermatids. Cell Rep.

[26] Monk D, Morales J, den Dunnen JT, Russo S, Court F, Prawitt D (2018). Recommendations for a nomenclature system for reporting methylation aberrations in imprinted domains. Epigenetics.

[27] Kerjean A, Dupont JM, Vasseur C, Le Tessier D, Cuisset L, Paldi A (2000). Establishment of the paternal methylation imprint of the human H19 and MEST/PEG1 genes during spermatogenesis. Hum Mol Genet.

[28] Marques CJ, João Pinho M, Carvalho F, Bièche I, Barros A, Sousa M (2011). DNA methylation imprinting marks and DNA methyltransferase expression in human spermatogenic cell stages. Epigenetics.

[29] Guo J, Grow EJ, Yi C, Mlcochova H, Maher GJ, Lindskog C (2017). Chromatin and Single-Cell RNA-Seq Profiling Reveal Dynamic Signaling and Metabolic Transitions during Human Spermatogonial Stem Cell Development. Cell Stem Cell.

[30] Langenstroth-Röwer D, Gromoll J, Wistuba J, Tröndle I, Laurentino S, Schlatt S, Neuhaus N (2017). De novo methylation in male germ cells of the common marmoset monkey occurs during postnatal development and is maintained in vitro. Epigenetics.

[31] Hammoud SS, Low DH, Yi C, Lee CL, Oatley JM, Payne CJ (2015). Transcription and imprinting dynamics in developing postnatal male germline stem cells. Genes Dev.

[32] Gaysinskaya V, Miller BF, de LC, van der Heijden GW, Hansen KD, Bortvin A (2018). Transient reduction of DNA methylation at the onset of meiosis in male mice. Epigenetics Chromatin.

[33] Gkountela S, Zhang KX, Shafiq TA, Liao WW, Hargan-Calvopina J, Chen PY, Clark AT (2015). DNA Demethylation Dynamics in the Human Prenatal Germline. Cell.

[34] Laurentino SS, Borgmann J, Gromoll J. On the origin of sperm epigenetic heterogeneity. Reproduction. 2016. 10.1530/REP-15-0436 26884419

[35] Benchaib M, Braun V, Ressnikof D, Lornage J, Durand P, Niveleau A, Guerin JF (2005). Influence of global sperm DNA methylation on IVF results. Hum Reprod.

[36] El Hajj N, Zechner U, Schneider E, Tresch A, Gromoll J, Hahn T (2011). Methylation status of imprinted genes and repetitive elements in sperm DNA from infertile males. Sex Dev.

[37] Laurentino S, Beygo J, Nordhoff V, Kliesch S, Wistuba J, Borgmann J (2015). Epigenetic germline mosaicism in infertile men. Hum Mol Genet.

[38] Navarro-Costa P, Nogueira P, Carvalho M, Leal F, Cordeiro I, Calhaz-Jorge C (2010). Incorrect DNA methylation of the DAZL promoter CpG island associates with defective human sperm. Hum Reprod.

[39] Richardson ME, Bleiziffer A, Tüttelmann F, Gromoll J, Wilkinson MF (2013). Epigenetic regulation of the RHOX homeobox gene cluster and its association with human male infertility. Hum Mol Genet.

[40] World Health Organization (2010). Department of Reproductive Health and Research. WHO laboratory manual for the examination and processing of human semen.

[41] Brinkworth MH, Weinbauer GF, Schlatt S, Nieschlag E (1995). Identification of male germ cells undergoing apoptosis in adult rats. J Reprod Fertil.

[42] Kossack N, Terwort N, Wistuba J, Ehmcke J, Schlatt S, Scholer H (2013). A combined approach facilitates the reliable detection of human spermatogonia in vitro. Hum Reprod.

[43] Neuhaus N, Yoon J, Terwort N, Kliesch S, Seggewiss J, Huge A (2017). Single-cell gene expression analysis reveals diversity among human spermatogonia. Mol Hum Reprod.

[44] Borgmann J, Tüttelmann F, Dworniczak B, Röpke A, Song H-W, Kliesch S (2016). The human RHOX gene cluster: target genes and functional analysis of gene variants in infertile men. Hum Mol Genet.

[45] Satija R, Farrell JA, Gennert D, Schier AF, Regev A (2015). Spatial reconstruction of single-cell gene expression data. Nat Biotechnol.

[46] Beygo J, Ammerpohl O, Gritzan D, Heitmann M, Rademacher K, Richter J (2013). Deep bisulfite sequencing of aberrantly methylated Loci in a patient with multiple methylation defects. PLoS One.

[47] Beygo J, Citro V, Sparago A, de Crescenzo A, Cerrato F, Heitmann M (2013). The molecular function and clinical phenotype of partial deletions of the IGF2/H19 imprinting control region depends on the spatial arrangement of the remaining CTCF-binding sites. Hum Mol Genet.

[48] Rahmann S, Beygo J, Kanber D, Martin M, Horsthemke B, Buiting K (2013). Automated methylation analysis of amplicons from bisulfite flowgram sequencing. PeerJ PrePrints.

[49] Albert S, Wistuba J, Eildermann K, Ehmcke J, Schlatt S, Gromoll J, Kossack N (2012). Comparative marker analysis after isolation and culture of testicular cells from the immature marmoset. Cells Tissues Organs (Print).

[50] R Core Team (2016). R: A language and environment for statistical computing.

