# Supplementary material for: High-resolution analysis of germ cells from men with sex chromosomal aneuploidies reveals normal transcriptome but impaired imprinting
Source: Clin Epigenetics. 2019 Aug 28;11:127. doi: 10.1186/s13148-019-0720-3 (PMC6714305; doi:10.1186/s13148-019-0720-3)
Supplement: Supplementary file 1 — Figure S1. Relative gene expression data from human germ cell and somatic cell fractions compared to the initial testicular cell population. Figure S2. Phase contrast micrographic images of human testicular cell cultures. (DOCX 1491 kb) [file 13148_2019_720_MOESM1_ESM.docx]

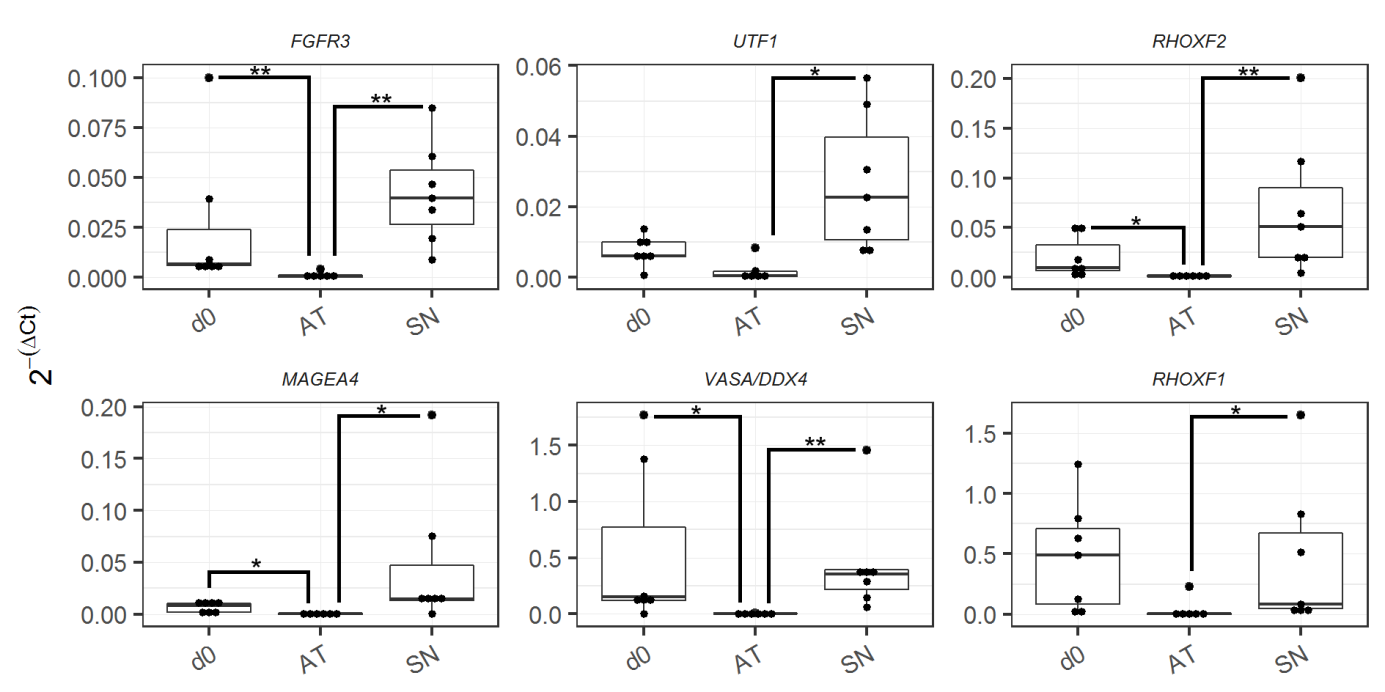
 Fig. S1: Relative gene expression data from human germ cell and somatic cell fractions compared to the initial testicular cell population. Relative gene expression levels are shown as 2^(–ΔCt)^ following normalization to the reference gene *GAPDH*. Significant differences are indicated as *p< 0.05 and **p<0.01. Abbreviations: AT = attached cell fraction, d0 = cell suspension collected prior to culture, SN = supernatant fraction.


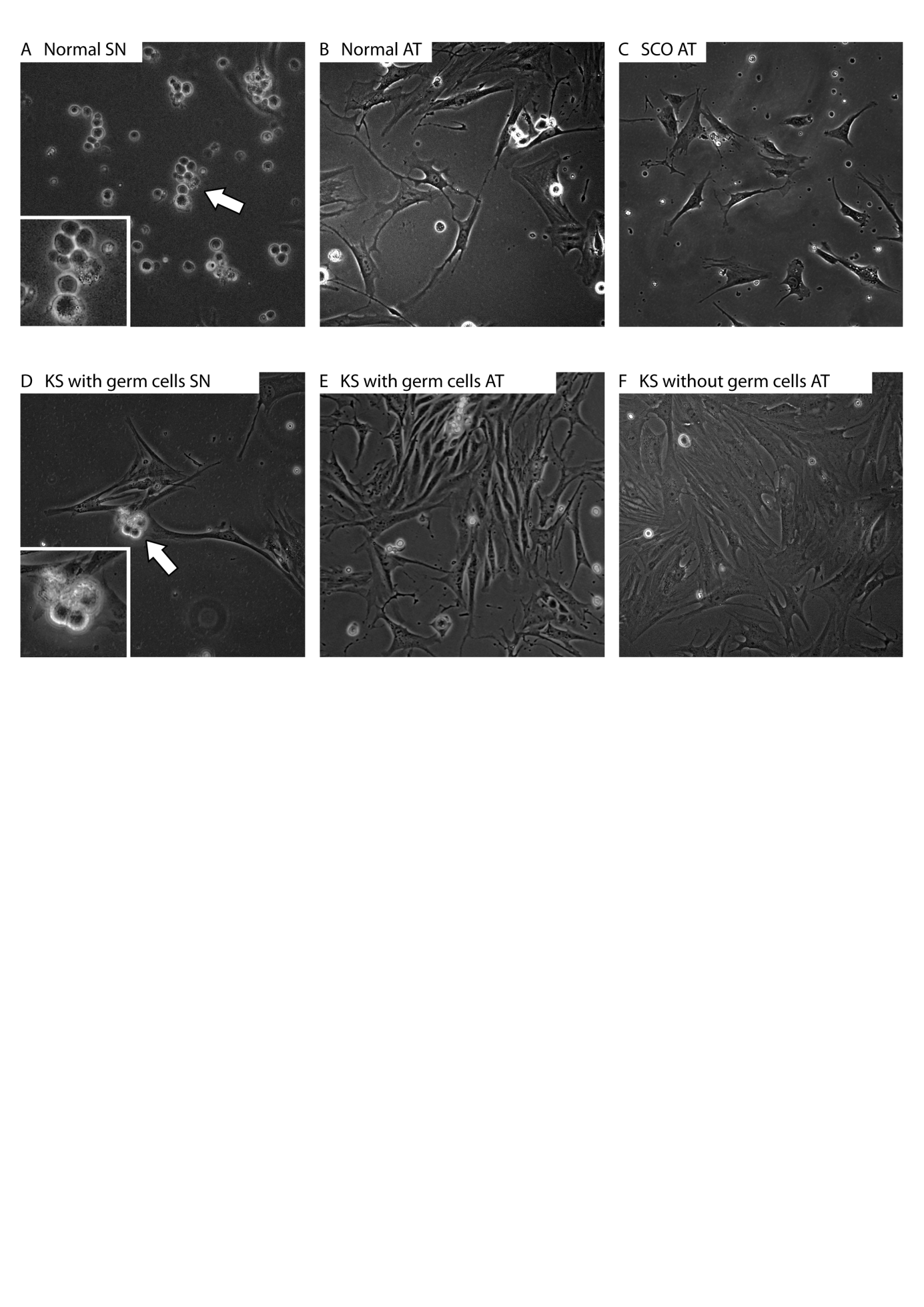
 Fig. S2: Phase contrast micrographic images of human testicular cell cultures. (A.,B.) Germ cell (SN) and somatic cell enriched (AT) fractions from patients with qualitatively normal spermatogenesis, Sertoli cell only syndrome (C.) and Klinefelter patients (D.-F.). Spermatogonial clusters are indicated by white arrows. Abbreviations: AT = attached cell fraction; SN = supernatant cell fraction; KS = Klinefelter syndrome; (+) = with germ cells; (-) = without germ cells. Scale bars represent 50 µm.
