# Supplementary material for: High-resolution analysis of germ cells from men with sex chromosomal aneuploidies reveals normal transcriptome but impaired imprinting
Source: Clin Epigenetics. 2019 Aug 28;11:127. doi: 10.1186/s13148-019-0720-3 (PMC6714305; doi:10.1186/s13148-019-0720-3)
Supplement: Supplementary file 2 — Table S1. Overview of patient samples included in this study and analyses to which they were subjected. Table S2. Endocrine parameters of the patient samples. Table S3. Gene names and primer sequences for transcript expression (using quantitative PCR) of selected germ cell marker genes. Table S4. Gene names and primer sequences for DNA methylation analysis (by deep bisulfite sequencing) of imprinted genes, selected germ cell markers, and XIST. (DOCX 22 kb) [file 13148_2019_720_MOESM2_ESM.docx]

**Table S1: Overview of patient samples included in this study and analyses to which they were subjected.**

|  | **Histological Analysis** | **qPCR for germ cell marker genes** | **Single cell RNA-Seq** | **Methylation analyses** |
| --- | --- | --- | --- | --- |
| **Blood** |  |  |  |  |
| Normal |  |  |  | n=5 |
| KS |  |  |  | n=7 |
| **Sperm** (Normozoospermic) |  |  |  | n=6 |
| **Testicular tissues** |  |  |  |  |
| Normal spermatogenesis | n=8 | n=8 |  | n=5 |
| Sertoli cell only | n=3 |  |  | n=3 |
| KS | n=7 |  | n=1 | n=6 |

Abbreviation: KS = Klinefelter syndrome

**Table S2: Endrocrine parameters of the patient samples.**

| **Patients** | **Endocrine parameters** | | |
| --- | --- | --- | --- |
|  | **FSH** (1-7 U/l) | **LH** (2-10 U/l) | **Testosterone** (>12 nmol/l) |
| **Normal** (n=5) |  |  |  |
| 1 | 3.8 | 2.7 | 25.9 |
| 2 | 5.6 | 2.3 | 17.9 |
| 3 | 2 | 3.4 | 13.2 |
| 4 | 10.8 | 2.5 | 15.7 |
| 5 | 4.4 | 5.9 | 10.3 |
| **SCO** (n=3) |  |  |  |
| 1 | 25.2 | 5.9 | 23.9 |
| 2 | 20.7 | 8.9 | 14.9 |
| 3 | 25.2 | 5 | 15.8 |
| **KS +** (n=4) |  |  |  |
| 1 | 93.6 | 29.4 | 15.4 |
| 2 | 7.3 | 3.7 | 14.8 |
| 3 | 15.1 | 5.7 | 14 |
| 4 | 6.9 | 3.2 | 15.3 |
| **KS -** (n=3) |  |  |  |
| 1 | 25.8 | 12.6 | 23.5 |
| 2 | 54.2 | 19.2 | 9.3 |
| 3 | 18.2 | 5.9 | 13 |

Abbreviations: FSH = Follicle stimulating hormone; LH = Luteinizing hormone; Normal = testicular tissues with qualitatively normal spermatogenesis; SCO = Sertoli cell only syndrome; KS = Klinefelter syndrome; (+) = with germ cells; (-) without germ cells.

**Table S3: Gene names and primer sequences for transcript expression (using quantitative PCR) of selected germ cell marker genes.**

| **Gene names** | **Primer orientation and sequences** |  |
| --- | --- | --- |
| *FGFR3 (Fibroblast growth factor receptor 3)* | 5’-GCGTACTGTGCCACTTCAGTGT-3’  3’-TCCTCCCCGTCTTCGTCAT-5’ |  |
| *MAGE A4 (MAGE family member A4)* | 5’-GGAGCACCAAGGAGAAGATCTG-3’  3'-AGGCAAGAGTGCAGGCAAA-5’ |  |
| *UTF1 (Undifferentiated embryonic cell transcription factor 1)* | 5’-ACGAGCAGATCCGGAAGCT-3’  3’-GGCGAGGCCGTTTGC-5’ |  |
| *RHOXF1 (Rhox homeobox family member 1)* | 5’-CTTGCCGAAAACTTAGGTGTGA-3’  3’-CGCCTACATCTGGCCCTTT-5’ |  |
| *RHOXF2/2B (Rhox homeobox family member 2*) | 5’-CGAGCAGTTCCCCAGTGAGT-3’  3’-CGAGTTCAGTCACATTCATGCTT-5’ |  |
| *DDX4 (DEAD (Asp-Glu-Ala-Asp) box polypeptide 4, VASA)* | 5’-GCCTCTGGGCGGAATTTT-3’  3’-CCATTGTGGATGTATTATCTCGCTTA-5’ |  |

**Table S4: Gene names and primer sequences for DNA methylation analysis (by deep bisulfite sequencing) of imprinted genes, selected germ cell markers, and XIST.**

| *Region name* | Primer sequence (with adaptors) |
| --- | --- |
| *KCNQ1OT1:*TSS-DMR (LIT1) | Fwd: 5’- CTTGCTTCCTGGCACGAG-  TTTATAGGTTTTTATATYGAGGGTTTATAGTAG -3’  Rev: 5’- CAGGAAACAGCTATGAC-  AAATAAACYRAAAACACRAACCAATTCTCTAC -3’ |
| *MEG3:*TSS-DMR | Fwd: 5’- CTTGCTTCCTGGCACGAG-  AAGAGGGAATAGTTTTGAGATTTTT -3’  Rev: 5’- CAGGAAACAGCTATGAC-  TAACCCCTCACTAACCTTATCACA -3’ |
| *H19/IGF2:*IG-DMR | Fwd: 5’- CTTGCTTCCTGGCACGAG-  GGTAYGGAATTGGTTGTAGTTGTGG -3’  Rev: 5’- CAGGAAACAGCTATGAC-  ATATCCTATTCCCAAATAACCCC -3’ |
| *MEST:*alt-TSS-DMR | Fwd: 5’- CTTGCTTCCTGGCACGAG-  TYGTTGTTGGTTAGTTTTGTAYGGTT -3’  Rev: 5’- CAGGAAACAGCTATGAC-  CCAACCACACCCCCTCRTTCCCAC -3’ |
| *FGFR3* | Fwd: 5’- CTTGCTTCCTGGCACGAG-  TTTTTTTTGTGAGGGTGGATATAG -3’  Rev: 5’- CAGGAAACAGCTATGAC-  AACACAAACCTAAAACTAACTACCTACC -3’ |
| *DDX4/VASA* | Fwd: 5’- CTTGCTTCCTGGCACGAG-  TTTTGTATTTATAGGTTTAATAGGTTATTT -3’  Rev: 5’- CAGGAAACAGCTATGAC-  TCCACACTTTAACCAAAAATC -3’ |
| *RHOXF1* | Fwd: 5’- CTTGCTTCCTGGCACGAG-  TGAGAATAGGAATTAGTTTTATTTTT -3’  Rev: 5’- CAGGAAACAGCTATGAC-  CCTTAACCAACATAACCTTCTAC -3’ |
| *XIST* | Fwd: 5’- CTTGCTTCCTGGCACGAG-  TGGGTTGTTGTATTTTTTGGAATAT -3’  Rev: 5’- CAGGAAACAGCTATGAC-  ACCTAACCTACTATCATCCATCTTACCT -3’ |
